# A comparative assessment of the morphology of *Profilicollis altmani* (Acanthocephala, Polymorphidae) from crustaceans and shore birds in Peru, with special notes on hook elemental analysis (EDXA), SEM imaging, histopathology, and molecular profile

**DOI:** 10.1051/parasite/2022005

**Published:** 2022-02-22

**Authors:** Omar M. Amin, Sara M. Rodríguez, Nataliya Rubtsova, Richard A. Heckmann, César Peña, Teresa Castro, Felipe Rivera, Guillermo D’Elía

**Affiliations:** 1 Institute of Parasitic Diseases 11445 E. Via Linda 2-419 Scottsdale AZ 85259 USA; 2 Instituto de Ciencias Marinas y Limnológicas, Facultad de Ciencias, Universidad Austral de Chile Isla Teja s/n Valdivia 509000 Chile; 3 Department of Biology, Brigham Young University Provo UT 84602 USA; 4 Departamento de Acuicultura, Facultad de Oceanografía y Pesquería, Universidad Nacional Federico Villarreal Francia 726 Miraflores Lima 15007 Perú; 5 Laboratorio de Patobiología Acuática, Dirección General de Investigaciones en Acuicultura, Instituto del Mar del Perú (IMARPE) Esquina Gamarra y General Valle s/n. Casilla Postal 22 Callao Perú; 6 Electron Microscopy Facility, Brigham Young University Provo UT 84602 USA; 7 Instituto de Ciencias Ambientales y Evolutivas, Facultad de Ciencias, Universidad Austral de Chile, Valdivia Isla Teja s/n Valdivia 509000 Chile

**Keywords:** Acanthocephala, *Profilicollis altmani* cystacanths, *Emerita analoga*, Adults, *Larus belcheri*, Peru, Descriptions, EDXA, Molecular profile, Histopathology

## Abstract

The morphology of cystacanths and adults of *Profilicollis altmani* (Perry, 1942) Van Cleave, 1947 (Polymorphidae) were studied from the Pacific mole crab *Emerita analoga* (Stimpson) (Crustacea, Hippidae) and Belcher’s gull *Larus belcheri* (Vigors) (Aves, Laridae), respectively, in Peru. Comparative morphometrics with accounts of other populations of *P. altmani* from elsewhere off the Pacific and Atlantic coasts of North and South America revealed marked intraspecific population variations. We report scanning electron micrographs (SEM) of new features, not before noted or captured in line drawings by earlier observers. We further present microscope images that reveal internal details not previously reported or possible to see with SEM. Energy dispersive X-ray analysis (EDXA) revealed unusual patterns in the chemistry of proboscis hooks especially the high sulfur and diminished phosphorous and calcium in hook tips and low sulfur and high levels of phosphorous and calcium at mid hooks. The size and shape of all hooks of the cystacanths are reported for the first time. Histopathological studies in *L. belcheri* from Peru are also included. Cystacanths of *P. altmani* from California were also analyzed for molecular patterns and compared with other sequences reported from other locations. The molecular data and the analysis of our new sequences of cytochrome oxidase I (COI) showed that haplotypes of *P. altmani* had low genetic variation; the species is not geographically structured, and within its clade no monophyletic group is formed.

## Abstract (fr)

La morphologie des cystacanthes et des adultes de *Profilicollis altmani* (Perry, 1942) Van Cleave, 1947 (Polymorphidae) a été étudiée, respectivement, à partir du crustacé *Emerita analoga* (Stimpson) (Crustacea, Hippidae) et du Goéland Siméon *Larus belcheri* (Vigors) (Aves, Laridae), au Pérou. La morphométrie comparative avec des données d’autres populations de *P. altmani* d’autres localités au large des côtes du Pacifique et de l’Atlantique de l’Amérique du Nord et du Sud a révélé des variations intraspécifiques marquées des populations. Nous rapportons des nouvelles observations de microscopie électronique à balayage, non notées auparavant ou non capturées dans des dessins au trait par des observateurs antérieurs. Nous présentons en outre des images au microscope qui révèlent des détails internes qui n’ont pas été signalés auparavant ni possibles à voir avec MET. L’analyse aux rayons X à dispersion d’énergie (EDXA) a révélé des modèles inhabituels dans la chimie des crochets de la trompe, en particulier la teneur élevée en soufre et une diminution du phosphore et du calcium dans les pointes des crochets et des niveaux faibles en soufre et en phosphore et en calcium au milieu des crochets. La taille et la forme de tous les crochets des cystacanthes sont décrites pour la première fois. Des études histopathologiques sur *L. belcheri* du Pérou sont également incluses. Les cystacanthes de *P. altmani* de Californie ont également été analysés pour les modèles moléculaires et comparés à d’autres séquences rapportées d’autres endroits. Les données moléculaires et l’analyse de nos nouvelles séquences de cytochrome oxydase I (COI) ont montré que les haplotypes de *P. altmani* avaient une faible variation génétique. L’espèce n’est pas structurée géographiquement, et au sein de son clade aucun groupe monophylétique n’est formé.

## Introduction

Cystacanths and adults of *Profilicollis altmani* (Perry, 1942) Van Cleave, 1947 (Polymorphidae) are common parasites of mole crabs and shore birds, respectively, along the Pacific and Atlantic coasts of North and South America [33, 53, 57, 59]. The acanthocephalan was first described as *Filicollis altmani* Perry, 1942 from specimens obtained from surf scoters (*Melanitta perspicillata* Linn. and *M. deglandi* Bonaparte) at Carmel Bay, California, USA by Perry [54]. It was later described as *Polymorphus kenti* Van Cleave, 1947 from a herring gull (*Larus argentatus* Pontoppidan) at New Brunswick, Canada by Van Cleave [75]. Shortly thereafter, it was described as *Polymorphus texensis* from the sanderling (*Crocethia alba* Pallas) at Galveston Island, Texas, USA by Webster [77]. In Peru, the same species was described as *Polymorphus* (*Profilicollis*) *bullocki* Mateo, Córdova, Guzmán, 1982 from *Larus belcheri* Vigors off the Lima coast by Mateo et al. [47]. Gomez-Puerta and Naupay [31] later reported 1 male specimen of *P. altmani* from the same host species and same location. Our Peruvian adult specimens and cystacanths were made available to Omar Amin by César Peña while on a conference tour in Peru in 2009 as whole mounts, and some cystacanths that were in ethanol were used to generate SEM images in 2015. Karl [42] gave an emended description of adults, as *Polymorphus altmani*, from 8 species of shorebirds and described the cystacanths from the mole crab *Emerita analoga* (Stimpson) (Hippidae) along the coast of California. Many authors treated these synonyms as independent species and some like Petrochenko [55], as *Parafilicollis* Petrochenko, 1956), Hoklova [40], and Amin [3] have included them in dichotomous keys based on intraspecific variations in proboscis armature and length of proboscis. We found that Karl’s [42] data from about 2000 specimens of *P. altmani* that he studied covered the full range of proboscis measurements and armature known for all 4 synonyms. Two or more species have been recognized as synonyms by Karl [42], Tantaleán and Cárdenas [69], Tantaleán et al. [70], and Nickol et al. [53], among more recent observers. Nickol et al. [52] reintroduced *Profilicollis* as a genus in the Acanthocephala. Amin [5] provided the definitive list of synonymies as follows: “***P. altmani*** (Perry, 1942) Van Cleave, 1947 [syns. *Filicollis altmani* Perry, 1942; *Parafilicollis altmani* (Perry, 1942) Petrochenko, 1956; *Polymorphus bullocki* Mateo, Cordova et Guzman, 1982; *Profilicollis kenti* (Van Cleave, 1947) Khokhlova, 1974; *Polymorphus kenti* Van Cleave, 1947; *Parafilicollis kenti* (Van Cleave, 1947) Petrochenko, 1956; *Falsificollis kenti* (Van Cleave, 1947) Yamaguti, 1963 *fide* Nickol et al. [53]; *Filicollis sphaerocephalus* sensu Harrington et Pillbury, 1938 *fide* Tantaleán et al. [71]; *Profilicollis texensis* (Webster, 1948) Khokhlova, 1974; *Polymorphus* (*Falsificollis*) *texensis* (Webster, 1948) Yamaguti, 1963 *fide* Nickol et al. [53].”

Most reports of *P. altmani* are known from the Pacific coast of North and South America (Tables 1, 3). A few reports are also known off the Atlantic coast of North America and South America. For instance, Nickol et al. [53] described cystacanths of *P. altmani* from the mole crab, *Emerita talpoida* Say from Atlantic Beach, North Carolina, USA. Bullock in Amin [4] identified *Polymorphus* (*Profilicollis*) *kenti* (= *P. altmani*) cystacanths from an unidentified species of *Emerita* in Florida, USA and he (Bullock in a pers. Communication to Karl [42]) found cystacanths identified by Karl as *P. altmani* in a species of *Emeritus* from Florida. Adults were also identified off the Atlantic from “willets and sanderlings in Louisiana and Mississippi” [53]. In South America, cystacanths of *P. altmani* were also reported from *Emerita brasiliensis* off Atlantic Uruguay [57]. Constancio [25] examined the prevalence and intensity of *P. altmani* infecting *E. analoga* from Pismo Beach, California and studied the effects of season and infections on the carotenoid concentration and composition on the mole crab. Most of the host related environmental work pertaining to this species, was, however, reported from Pacific South America. In Chile, sea birds arriving in mass numbers during the summer enhance the spread of acanthocephalan eggs to the environment, producing higher values of infection in *E. analoga* [58, 80]. The identity of definitive host species was found to be relevant to morphometric and reproductive variations (fecundity) of *P. altmani* with acanthocephalans of 2 species of *Larus* attaining larger body size than those in 2 other genera of gulls from the Chilean coast [56]. Iannacone et al. [41] reported a prevalence of 55.3% and a mean intensity of 2.21 of *P. altmani* cystacanths in a sample of 860 specimens of *E. analoga* from Chorrillos fish market, Lima, Peru; host sex and size were unrelated.

**Table 1:** Morphometrics of cystacanths of *Profilicollis altmani* from the USA, Peru, Chile, and Uruguay.

Study site	USA		Peru		Chile	Uruguay
	California Pacific	North Carolina Atlantic	Chorrillos, Lima Pacific	Playa de Pasamayo, Lima Pacific	Lenga Pacific	Arachaña beach Atlantic
Host	*Emerita analoga*	*Emerita. talpoida*	*Emerita analoga*	*Emerita analoga*	*Emerita analoga*	*Emerita brasilensis*
Source	Karl (1967)	Nickol et al. (2002)	This study	Mateo et al. (1983)	Balboa et al. (2009)	Rodríguez & D’Elía (2016)
Total body length*	5300–7810 (750)**	3986–6017 (4949)	4525–6675 (5555)	5500–7500***	5000–6500 (5700)	4500–6200 (5533)
Maximum body width	260–400 (360)	624–845 (663)	750–975 (884)	890–1130	470–1490 (1143)	660–915 (755)
Proboscis						
Shape	Ovoid	Ovoid	Ovoid	Ovoid	Ovoid	Ovoid
Length	750–800 (Figs. 18, 20)	518–648 (571)	416–728 (632)	450–600 (550)	500–900 (696)	505–730 (608)
Width	400–500 (Figs. 18, 20)	230–364 (269)	250–458 (352)	260–390 (330)	329–600 (456)	383–610 (485)
Rows of hooks	21–35 (7)	28	26–34 (29)	27–33	26–30	26–29
Hook/row	10–15 (13)	12	13–16 (14.5)	13–15	14–16	14–15
Length of hooks						
Apical	30–50	34–43 (37)	35–45 (38)	42	56	41–48 (44)
Medial	30–60	41–53 (45)	35–43 (39)	35	73	48–61 (54)
Basal	50–70	43–57 (52)	50–60 (55)	53	90	52–79 (64)
Proboscis receptacle						
Length	1720–2860 (2380)	1718–2026 (1868)	1775–2900 (2309)	1870–3000 (2380)	1400–2970 (2562)	1600–2010 (1783)
Width	220–410 (230)	–	200–400 (294)	–	240–470 (338)	318–450 (369)
Neck						
Length	1140–1830 (1700)	1000–1740 (1512)	950–1600 (1180)	1000–1600 (1210)	850–1690 (1232)	805–1100 (939)
Width	290–470	336–470 (406)	250–425 (361)	–	350–580 (473)	379–595 (461)
Lemnisci						
Length	Variable unequal	1037–1440 (1250)	925–2400 (1580)	1300–1900 (1550)	1290–2000 (1642)	–
Width	–	–	62–250 (155)	–	100–340 (181)	–

* Total body length includes the proboscis, neck, and the 3 trunk regions. Specimens with retracted tail are not used. Measurements of each developing trunk region and incipient testes are not included separately because of their extreme variability in the growing cystacanths.

** Range (mean) in micrometers in all measurements.

*** Described as *Polymorphus* (*Profilicollis*) *bulloki*.

*Profilicollis altmani* can also successfully infect mammals with ensuing potentially serious outcomes, which may occasionally represent a public health hazard. Worms introduced to rats, mice, hamsters, and pups have produced viable infections detectable in the body cavity and the walls of the small and large intestines. There is potential for risk for humans who eat infected sand crabs [70]. “Muy muy” (*E. analoga*) heavily infected with cystacanths consumed by humans in various forms on the southern beaches of Lima have been evaluated as a cause of a potential public health problem among 500 people surveyed [50]. Likewise, domestic dogs were reported foraging on mole crabs at Curiñanco beach, southern Chile, which represents a potential health risk for the dogs [61]. In California, morbidity and mortality of the southern sea otter, *Enhydra lutris nereis* (Linn.) were attributed to massive infections with *P. altmani* reaching 8760 worms per animal causing intestinal perforations, nutrient depletion, and mortality [49, 63]. Histopathological lesions in the intestine of another definitive host in Peru, the grey gull *Leucophaeus modestus* (Tschudi) were explored by Gonzales-Viera et al. [32].

The following are perspectives on available molecular findings of populations of *P. altmani* in South and North American Atlantic and Pacific coasts. Sequences of cystacanths of *P. altmani* collected from *Emerita brasiliensis* Schmitt in Uruguay produced 3 distinct haplotypes, suggesting that the “cystacanths recovered from *E. brasiliensis* on the southern Atlantic coast have low genetic variation and therefore belong to the same species, *P. altmani*, that has already been recorded on the Californian and Chilean Pacific coasts, as well as on the Atlantic coast of North America” [57]. Rodriguez et al. [59] concluded that haplotypes of *P. altmani* from Chile did not form a monophyletic group and clarified the role of environmental factors and host foraging behavior as determinants of predator-prey relationships. Goulding and Cohen [33] noted that sequences from COI and ITS loci revealed shared haplotypes between the North American east and west coast populations, indicating wide dispersal of this parasite.

Our work will contribute to the body of work that has been already reported by other observers of this acanthocephalan already known to have a wide host use and potential high impact on animal and even human populations. We shall supplement known morphology of this parasite with observations of newly observed structures using scanning electron micrographs and color optical micrographs, provide comparative morphometrical information, analyze the chemistry of hooks and spines for the first time, produce new molecular analysis from cystacanths from Peru, and produce new histopathological images from *L. belcheri* for the first time.

## Materials and methods

### Collections

Specimens were deposited in the University of Nebraska’s State Museum’s Harold W. Manter Laboratory (HWML) collection, Lincoln, Nebraska, USA.

### Cystacanths

In the spring (November to December) of 1976, about 200 specimens of the Pacific mole crab, *Emerita analoga* were collected in Playa Pescadores, Chorrillos (12°10′00″ S – 77°02′00″ W), Lima, Peru and fixed in ethanol. The prevalence was 80% and intensity was from 2 (in small crustaceans) to 20–30 (in large crustaceans). In the spring (September to October) of 1982; 558 specimens of *E. analoga* were collected from Playa Conchán, Lurin (12°14′59″ S – 76°56′15″ W), Lima. The prevalence was 65% and the intensity ranged from 1 (in small crustaceans) to 9 parasites (in large crustaceans).

### Adults

In the spring (end of August) of 1981, 4 adult gulls, *Larus belcheri* from Pasamayo Beach (11°49′41″ S – 77°7′51″ W) were autopsied at the Ichthyology Laboratory (FOPCA-UNFV). All the birds were heavily parasitized and it was difficult to extract the parasites from the packed intestine. In the spring (September) of 1982, 2 adult *L. belcheri* were captured alive at Punta Roquitas Beach, in the Miraflores District, Lima (12°07′03″ S – 77°02′35″ W) and were used to carry out experimental infections with 50 cystacanths each that yielded successful infections with juvenile and adult specimens of *P. altmani.*

Twenty-four cystacanths were whole mounted on slides that were made available for microscopical studies. Twenty cystacanths (13 males, 7 females) were studied and measured. The specimens were all fixed in ethanol and stained with Borax carmine. A total of 42 adults of *P. altmani* were collected from 4 Belcher’s gulls, *L. belcheri* in Playa Pasamayo, Ancón (11°49′41″ S, 77°7′51″ W), Lima northern beach, Peru, during 1981–1982. Sixteen Borax carmine-stained whole mounted specimens were made available and 5 males and 11 females were studied and measured, and the remaining were processed for microscopical studies to confirm species identification.

The Peruvian material also included 8 slides of hematoxylin-eosin-stained histopathological sections of adult specimens of *P. altmani* in *L. belcheri* intestinal sections that were initially fixed in formalin; 2 slides from each of the 4 heavily infected gulls in the spring of 1981 noted above. The edges of some slides showed signs of crystallization.

A similar collection of 10 cystacanths of *Profilicollis botulus* Van Cleave, 1916 were gathered from the lined shore crab, *Pachygrapsus crassiceps* Randall at Anaheim Beach, California (33°41′34″ N – 118°0′1″ W), of which 5 specimens were sequenced to include in a phylogenetic analysis; remaining specimens were processed for microscopical confirmation of species identification. From California, about 10 cystacanths of *P. altmani* were collected from the body cavity of specimens of *E. analoga* at Redondo Beach (33°51′29″ N – 118°22′44″ W). Five specimens were used for molecular studies.

Genetic comparison and phylogenetic analyses were based on a fragment of 645 bp of the mitochondrial cytochrome oxidase I (COI) gene of one sequence of a *P. altmani* specimen*.* Details of these analyses are provided in Supplementary Material. Briefly, the new COI sequence was aligned to a matrix with one representative of each haplotypic class of *Profilicollis altmani, P. chasmagnathi*, *P. novaezelandensis*, and *P. botulus* from diverse intermediate and definitive hosts [29, 30, 33, 34, 46, 57, 59, 60] available in GenBank. As such, a total of 24 sequences of *Profilicollis* were analyzed (Table S1). In addition, sequences of *Polymorphus minutus* and *Arhythmorhynchus brevis* were used to form the outgroup. Sequence alignment and observed genetic distances (*p*) were done and calculated in MEGA 7 [68]. Finally, two methods of phylogenetic inference were implemented, maximum likelihood (ML), which was conducted with IQ-TREE, and Bayesian inference (BI) conducted with MrBayes 3.1 [62, 72] (see details in Supplemental Material).

### Comparative material

**Figures 1–4:**
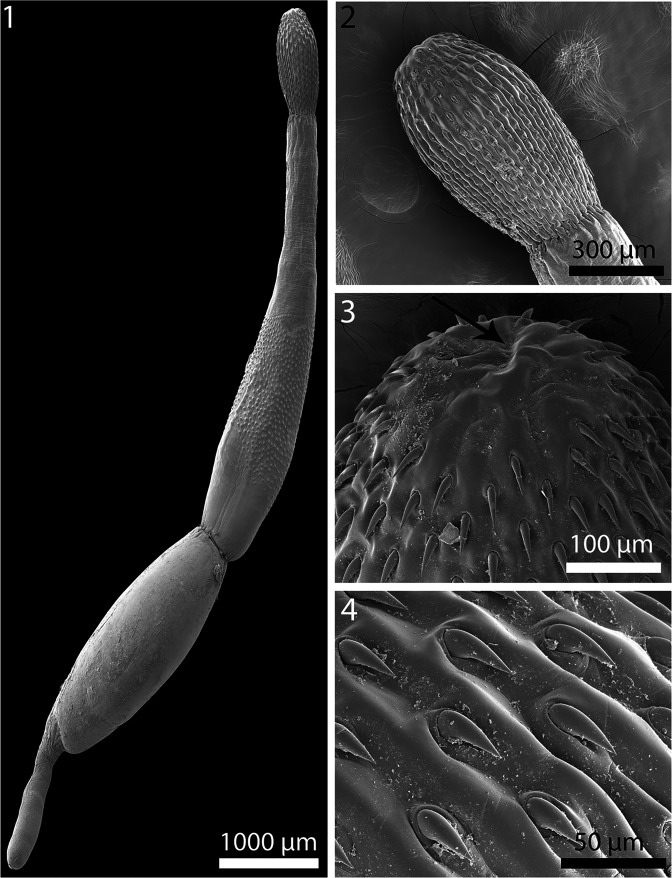
SEM of cystacanths of *Profilicollis altmani* from *Emerita analoga* on the Peruvian coastline, Lima. **1.** Entire female cystacanth showing proboscis, neck and trunk regions. **2.** Entire proboscis. **3.** An apical view of proboscis with external evidence of an apical organ (arrow). **4.** Mid-part of proboscis with hooks embedded in furrows.

**Figures 5–10:**
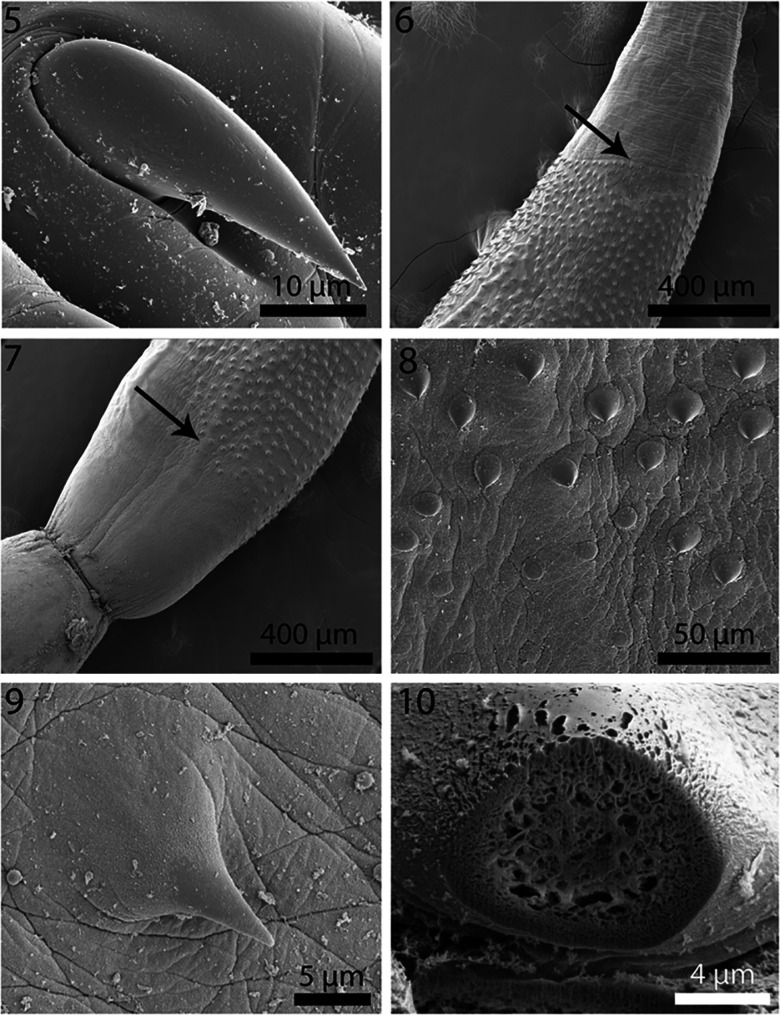
SEM of cystacanths of *Profilicollis altmani* from *Emerita analoga* on the Peruvian coastline, Lima. **5.** A robust hook in the mid-section of a proboscis with thick base. **6.** The posterior part of the neck interface with the anterior spinose trunk of a cystacanth. Note anterior spines in perfect ring (arrow). **7.** The posterior part of anterior trunk section showing the incomplete spination posteriorly (arrow). **8.** More posterior trunk spines are occasionally in irregular circles randomly distributed. **9.** A high magnification of a trunk spine showing cross serrations of cuticular surface. **10.** A Gallium cut section of a trunk spine showing its spongy center and denser cortical layer.

**Figures 11–16:**
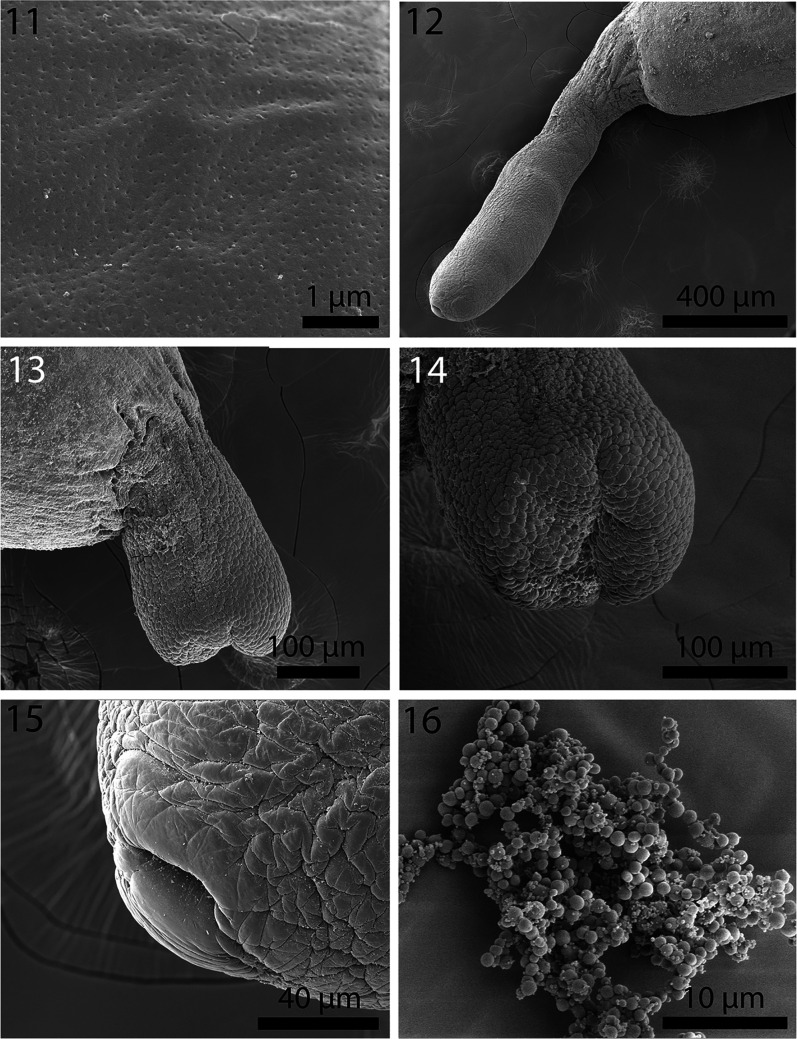
SEM of cystacanths of *Profilicollis altmani* from *Emerita analoga* on the Peruvian coastline, Lima. **11.** Micropores from the mid-trunk region. **12.** A fully extended tail. **13.** Lateral view of a partially retracted tail. **14.** Posterior view of the partially retracted tail showing invagination point and the coarse corrugated texture of the cuticular surface. **15.** Genital orifice of a female gonopore at the posterior end of a fully extended tail. Note the thick un-corrugated lips. **16.** A cluster of developing eggs in a female cystacanth.

Figures in all previous descriptive accounts were only created in line drawings that we are not duplicating. We are referring interested parties to original line-drawings in the following descriptions. The first description by Perry [54] (page 387) included 4 line-drawings of a whole male with spherical testes, anterior end of a female, part of a middle section of a proboscis not showing hook roots, and an egg. Van Cleave’s [75] description of *P. kenti*, included 2 line-drawings of a praesoma and of half a proboscis not showing hook roots (pages 305, 309). Webster’s [77] description of *P. texensis* included 6 line-drawings (page 67) of a male, a female, a few rooted hooks, spines, and 2 eggs. In their description of *P. bullocki*, Mateo et al. [47] provided line drawings in 5 figures of a male, female reproductive system, rooted hooks nos. 1, 4, 5, 14, and eggs (page 5). Mateo et al. [48] also included 2 line-drawings of a male cystacanth and a proboscis. Karl [42] provided many line-drawings of males and females (Figs. 1–8, page 83–87), developing proboscides (Figs. 9–11, page 89), young adults (Figs. 22–23, page 99–101), and male and female cystacanths (Figs. 18, 21, page 95–97). All line-drawings of this acanthocephalan species by other and subsequent observers are only renditions of one or more in the above quoted accounts.

**Figures 17–22:**
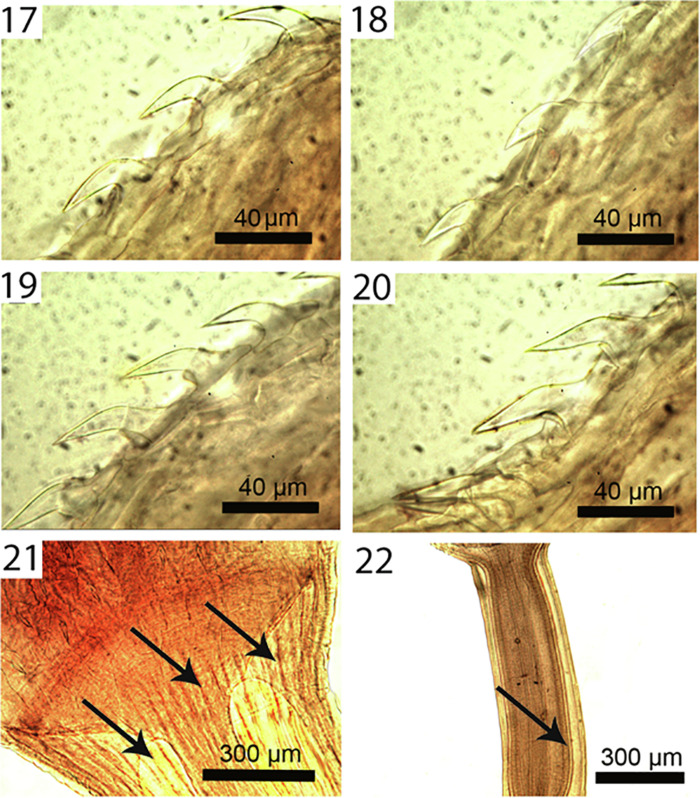
Micrographs of cystacanths and adults of *Profilicollis altmani* from *Emerita analoga* and from *Larus belcheri*, respectively on the Peruvian coastline, Lima. **17–20.** Lateral views of hooks and roots of one longitudinal row on the proboscis of a cystacanth. **17.** Apical and 2 subapical hooks and roots. **18.** Hook nos. 4, 5, and 6 with their roots. Note the shorter and thickest hook no. 5 (at middle). **19.** Hook nos. 7–10; note the increasing size of hooks posteriorly. **20.** Hook nos. 11–15. Note the increasing size of hooks posteriorly being longest and more crowded basally. **21.** Fan shaped anterior end of proboscis receptacle inserted at the posterior end of the proboscis. Note the heavy muscular fibers of the neck (arrows). **22.** Double-walled proboscis receptacle (arrow) within the neck of an adult specimen directly posterior to attachment to proboscis.

**Figures 23–28:**
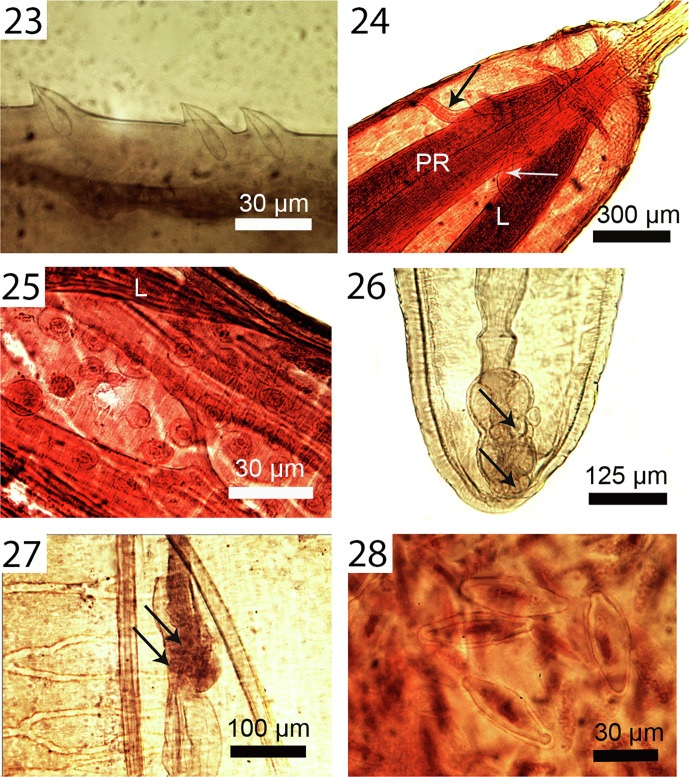
Micrographs of adult females of *Profilicollis altmani* from *Larus belcheri*, on the Peruvian coastline, Lima. **23.** Lateral view of trunk spines showing their single drop-shaped core support structure. **24.** Anterior end of a specimen showing the 2 lateral retinacular nerves (arrow), the proboscis receptacle (PR) and a lemniscus (L). **25.** Anterior trunk showing the prominent branching longitudinal muscle fibers and the hypodermic nucleated cells in the area of the fibrillar attachment of a lemniscus (L) to the body wall. **26.** Posterior part of a female reproductive system showing the near terminal position of the gonopore and the characteristic 4 horizontal nucleated cells at the posterior end of the vagina and between the sphincters (arrows). **27.** Simple, thin-walled uterine bell with few small nucleated uterine bell cells (arrows). Note part of the reticular lacunar system (left) and median ligaments. **28.** Eggs. Note the thick outer shell and the polar prolongation of the fertilization membrane.

### Optical microscope images

Optical microscope images were acquired using a BH2 light Olympus microscope (Olympus Optical Co., Osachi-shibamiya, Okaya, Nagano, Japan) attached to an AmScope 1000 video camera (United Scope LLC, dba AmScope, Irvine, CA, USA), linked to an ASUS laptop equipped with HDMI high-definition multimedia interface system (Taiwan–USA, Fremont, CA, USA). Images from the microscope are transferred from the laptop to a USB and stored for subsequent processing on a computer.

### Scanning electron microscopy (SEM)

About 15 specimens that had been fixed and stored in 70% ethanol were processed for SEM following standard methods [44]. These included critical point drying (CPD) (Tousimis Automandri 931.GL) and mounting on aluminium SEM sample mounts (stubs) using conductive double-sided carbon tape. Samples were sputter coated with an 80–20% gold-palladium target for 3 minutes using a sputter coater (Quorum (Q150T ES) www.quorumtech.com) equipped with a planetary stage, depositing an approximate thickness of 20 nm. Samples were placed and observed in an FEI Helios Dual Beam Nanolab 600 (FEI, Hillsboro, OR, USA) Scanning Electron Microscope (FEI). Samples were imaged using an accelerating voltage of 5 kV, and a probe current of 86 pA, at high vacumm using an SE detector.

### Focused Ion Beam (FIB) sectioning of hooks

A dual-beam SEM with gallium (Ga) ion source (GIS) was used for the LIMS (Liquid Ion Metal Source) part of the process. The gallium beam (LIMS) is a gas injection magnetron sputtering technique whereby the rate of cutting can be regulated. The hooks were sectioned at two positions (tip and middle) using the FEI Helios Dual Beam Nanolab mentioned above. The dual-beam FIB/SEM is equipped with a gallium (Ga) Liquid Ion Metal Source (LIMS). The hooks of the acanthocephalans were centered on the SEM stage and cross-sectioned using an ion accelerating voltage of 30 kV and a probe current of 2.7 nA, following the initial cut. The time of cutting is based on the nature and sensitivity of the tissue. The sample also goes through a cleaning cross-section milling process to obtain a smoother surface. The cut was analyzed with an X-ray normally at the tip, middle, and base of hooks for chemical ions with an electron beam (Tungsten) to obtain an X-ray spectrum. The intensity of the GIS was variable according to the nature of the material being cut. Results were stored with the attached imaging software then transferred to a USB for future use.

### Energy Dispersive X-ray analysis (EDXA)

The Helios Nanolab 600 is equipped with an EDXA (Mahwah, NJ, USA) TEAM Pegasus system with an Octane Plus detector. The sectioned cuts were analyzed by EDXA. Spectra of selected areas were collected from the center and the edge of each cross-section. EDXA spectra were collected using an accelerating voltage of 15 kV, and a probe current of 1.4 nA. Data collected included images of the displayed spectra as well as the raw collected data. Relative elemental percentages were generated by TEAM software.

### Histopathology

For the histological sections, heavily infected intestinal tissues of *L. belcheri* collected from Playa Pasamayo, Ancón, Lima in 1981 were fixed in 10% buffered formalin. After dehydration and embedding in paraffin, the specimens were processed using standard methods comparable to those of Kiernan [43] and Bancroft and Gamble [22]. These paraffin tissue blocks were sectioned at 4–6 microns, placed on glass slides and stained with hematoxylin and eosin (HE). The prepared glass slides were viewed with a BH2 light Olympus microscope (Olympus Optical Co., Osachi-shibamiya, Okaya, Nagano, Japan); see Microscope Images above.

## Results

The following treatment is based on the microscopic and SEM studies of cystacanths and adults of *P*. *altmani* collected from *E. analoga* and *L. belcheri*, at various beaches of Lima, Peru during 1976–1978 and 1981–1982, respectively. We note that the prevalence and intensity of infection of *E. analoga* with cystacanths decreased from 80% and 2–30 in 1976 to 65% and 1–9 in 1981. The microscopical study provided considerable new detail not known in the previous descriptions under the various names of this common acanthocephalan. The SEM studies added considerable new detail as this species was not previously studied with SEM.

The following parasite descriptions will be in 2 sections: cystacanths and adults.

## I Cystacanths

One descriptive account of cystacanths based on 7 cystacanths studied with SEM (Figs. 1–16) and on 20 cystacanths (13 males and 7 females) measured and studied with microscopic images (Figs. 17–36) from whole mounts obtained from *E. analoga* in Peru is provided below.

**Description of cystacanths from *E. analoga* from Peru.** (structures not previously reported are bolded)

With characters of the family Polymorphidae and genus *Profilicollis*. Body flattened dorso-ventrally, divided by constrictions into proboscis, long cylindrical neck, spinose trunk, ovoid hindbody, and tail of variable length that may be partially or wholly retracted (Fig. 1). See comparative measurements and counts in Table 1. Proboscis ovoid with flattened anterior end showing evidence of **apical organ** and indented longitudinal rows of at least 13 hooks each (Figs. 2–3). Hooks of similar shape (Fig. 4), increasing in length and diameter from apical to fourth hook. Fifth hook abruptly shorter but thickest, 6th hook, smallest; hooks gradually increasing in size posteriorly to maximum basally (Figs. 17–20). **Complete measurements of hooks in 1 longitudinal row in** **Table 2**. All hooks with gradually thickening base (Fig. 5), rooted with prominent anterior manubria (Figs. 17–20). **Hook tips with high level of sulfur and low levels of phosphorous and calcium and mid-hook showing opposite trend.** Neck long (Fig. 22) widening at base. Proboscis receptacle evident through neck extending to about half length of spinose trunk. **Trunk with many electron-dense micropores (****Fig. 11****) and incipient hypodermic nucleated cells (****Fig. 34****)**. Anterior trunk with irregular circles of spines ending anterior to trunk constriction (Fig. 8). **Spines pointed with broad base, dense cortical layer and spongy core (****Figs. 9, 10****). Anterior and middle spines with higher level of calcium than phosphorous and sulfur but posterior spines with low levels of all elements.** Lemnisci variable in length occasionally extending beyond or shorter than receptacle. **Posterior tail with corrugated texture**, often extended (Fig. 12) but occasionally partially retracted (Figs. 13, 14). Incipient testes spheroid to ovoid, often in spiny trunk region or occasionally in hind body (Fig. 34) where trunk may contain developing cement glands and barely distinguishable invaginated vestigial bursa. In female cystacanths, parts of developing reproductive system relatively visible (Fig. 35) with **proximal genital ducts developing as invagination of body wall (****Fig. 36****) and terminal gonopore with bulbous lips occasionally prominent (****Fig. 15****)** and **developing eggs occasionally observed (****Fig. 16****).**

**Figures 29–34:**
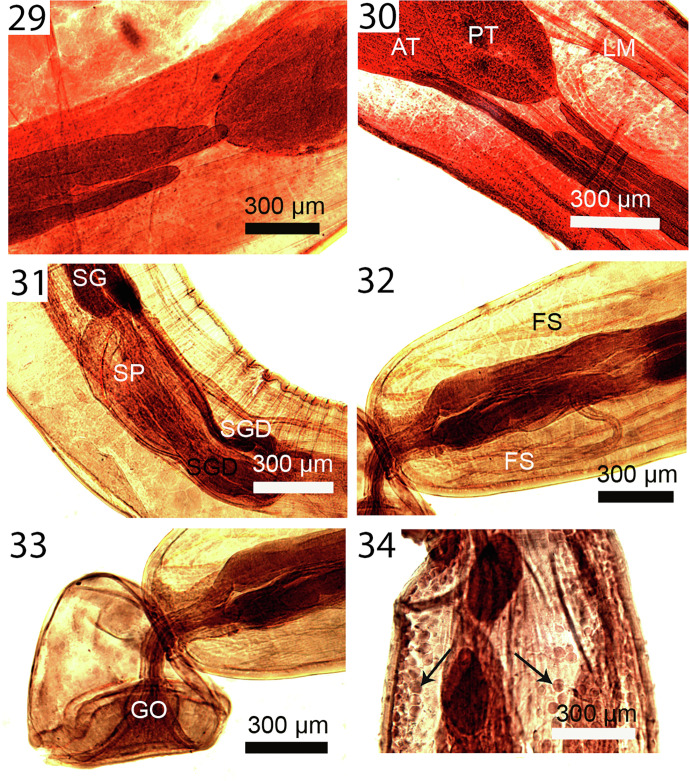
Micrographs of adult males of *Profilicollis altmani* from *Larus belcheri* (Figs. 29–33) and male cystacanth (Fig. 34) from *Emerita analoga* on the Peruvian coastline, Lima. **29.** Anterior end of the 4 tubular cement glands interfacing with the posterior testis. Note that the unequal cement glands do not extend equally anteriorly. **30.** Another example of extreme unequal anterior extension of cement glands to mid-point of posterior testis (PT) reaching anterior testis (AT). Note prominent longitudinal trunk muscles (LM). **31.** Posterior part of a male reproductive system showing posterior end of cement glands (CG), cement gland duct (CGD), and Saefftigen’s pouch (SP). **32.** A pre-bursal perspective showing independent cement glands’ ducts and associated fibral strands (FS). **33.** The bell-shaped bursa with the cement gland’s ducts channeling into the genital orifice (GO). No sensory structures were observed. **34.** Developing testes in a cystacanth posterior to the second body constriction. Note the early stages of the hypodermic cells (arrows) throughout that are more developed in adults in Figure 27.

**Figures 35–40:**
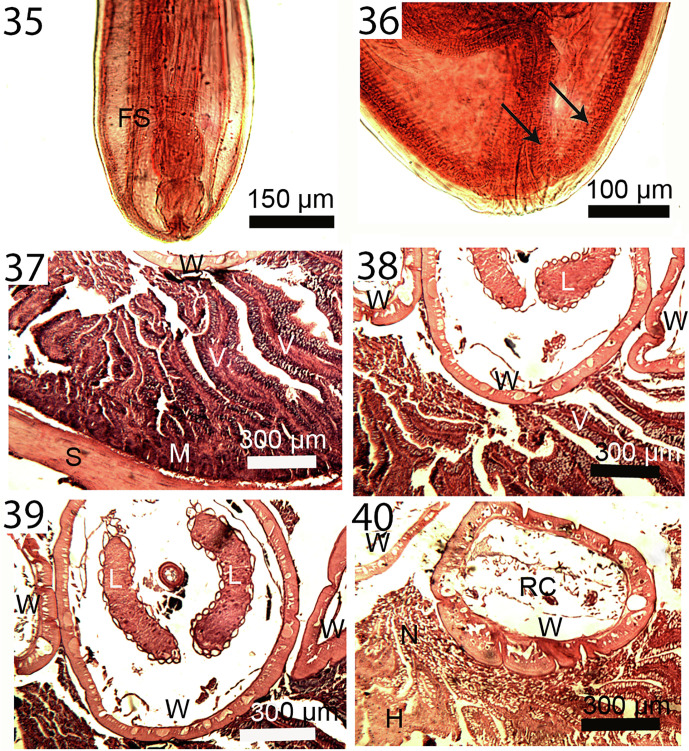
Micrographs of cystacanths (Figs. 35, 36) and adults in histopathological sections (Figs. 37–40) of *Profilicollis altmani* from *Emerita analoga* and *Larus belcheri*, respectively. **35.** Posterior portion of the incipient female reproductive system of a cystacanth. Note the two sets of lateral para-vaginal fibrillar strands; left bundle marked (FS). **36.** Terminal gonopore and the developing proximal portion of a female system in another cystacanth. Note the body wall layers invaginating into the barely visible reproductive system ducts (arrows). **37.** A section showing the serosa (S) of the host intestine and the mucosa and submucosa (M) with disruption of the villi (V) near the worm (W) interface. **38.** Crowding effect in this heavily infected host gut is evident with parts of these 3 worms (W) totally destroying the tips of the villi (V). The lemnisci (L) of the worm in the center are prominent. **39.** A different perspective of a section in another heavy infection with 3 worms (W) with the central worms featuring both lemnisci (L) with corrugated surface epithelium. Note the destruction of the tips of the villi (V) and its disintegration into necrotic tissue (N). **40.** A worm (W) in a different section of the host gut showing various types of destruction and disorientation of the mucosal layers, formation of necrotic tissue (N) and hemorrhaging (H). Reproductive cells (RC) continue to be produced in worms (females) (W) at that stage of infection.

**Table 2:** The size of 15 hooks in one longitudinal row on the proboscides of 10 cystacanths of *Profilicollis altmani* from *Emerita analoga* in Peru.

Hook no.	Hook length	Hook width at base
1	35–45 (38)*	9–14 (11)
2	37–45 (40)	11–16 (12)
3	39–45 (42)	12–17 (15)
4	38–45 (41)	14–17 (16)
5	30–45 (38)	15–20 (19)
6	21–40 (31)	10–18 (13)
7	30–40 (35)	10–16 (13)
8	35–45 (37)	10–19 (14)
9	35–50 (40)	10–20 (14)
10	37–50 (43)	12–20 (15)
11	40–57 (47)	11–20 (15)
12	42–57 (49)	12–19 (15)
13	42–58 (51)	12–19 (14)
14	45–57 (52)	11–19 (13)
15	50–60 (55)	10–19 (12)

* Range (mean) in micrometers.

### Taxonomic summary

Host: Pacific mole crab, *Emerita analoga* (Stimpson) (Crustacea: Hippidae).

Other hosts: See other hosts in Table 1.

Site of infection: Body cavity.

Locality: Playa Pescadores, Chorrillos (12°10′00″ S, 77°02′00″ W), Lima, Peru.

Other localities: See other localities in Table 1, including the Pacific and Atlantic coasts of North and South America.

Specimens: Cystacanths in HWML Parasitology Collection n. 216672.

### Remarks

Line-drawings of *P. altmani* cystacanths have rarely been presented except for those included in Karl [42]; our SEM and microscopic images show details presented here for the first time. Previously, only Nickol et al. [53] presented a photograph of the praesoma of a cystacanth and Balboa et al. [21] included an SEM of the apical end of a proboscis of the same species. Our newly observed details include all proboscis hooks and roots, trunk spine arrangement and anatomy, tail, emerging eggs and female gonopore. We also note the presence of micropores described here for the first time in cystacanths in any species of acanthocephalans; micropores have been previously described from adults only.

Our specimens had larger proboscides than in others reported from *E. analoga* in Peru by Mateo et al. [48] but smaller than those from the same host species in Chile by Balboa et al. [21] (Table 1). Our specimens had a rather wide variation in the number of hook rows (26–34) and number of hooks per row (13–16), respectively, relative to cystacanths in all other collections especially those from North Carolina (28 and 12) except those studied by Karl [42] who reported 21–35 rows of 10–15 hooks each (Table 1). The specimens from Chile and Uruguay had the largest size hooks compared to those from other collections in Peru and the United States. The proboscis receptacle was smallest in cystacanths from *E. brasiliensis* in Uruguay and those from *E. talpoida* in North Carolina. The lemnisci reached maximum length in our cystacanths from *E. analoga* in Peru with comparable average values in all collections. All above differences fall within the range of intraspecific variability and extremes may be related to host species and geography.

## II Adults

**Description of adults from *L. belcheri* in Peru** (structures not previously reported are bolded).

General. With character of Polymorphidae, genus *Profilicollis*. Shared structures larger in males than in females. Body elongate, cylindrical, divided by two mild constrictions into three distinct regions of about equal length, with the middle part being widest. See measurements and counts in Table 3. Proboscis spheroidal but occasionally oblate especially in older adults. Hooks, like those of cystacanths, similar in shape (Fig. 4) increasing in length and diameter from apical to fourth hook; fifth hook abruptly shorter but thickest, 6th hook, smallest, gradually increasing in size posteriorly to maximum basally (Table 2). All hooks rooted with prominent anterior manubria (see Figs. 17–20) as in cystacanths. Neck slender, somewhat cylindrical (Fig. 22) and widest posteriorly at junction with trunk. **Trunk walls with numerous small hypodermic nucleated cells (****Fig. 25****) and reticular lacunar system (****Fig. 27****).** Anterior trunk tapering towards the neck with complete circles of spines similar to those of cystacanths. **Spines pointed with broad base, elongate drop-shaped single-core rod support (****Fig. 23****) and denser cortical layer.** Posterior region of trunk tapers towards posterior end with terminal gonopores in both sexes. **Proboscis receptacle double-walled, fan-like anteriorly, arising at base of spheroid proboscis (****Fig. 21****), about twice as long as neck**. **Two prominent complex retinacular nerves emerging from cephalic ganglion slightly posterior to level of anterior trunk and passing through receptacle walls before entering into circular and longitudinal muscle layers of body wall (****Fig. 24****). Lemnisci (****Fig. 24****) not always equal and vary in length appearing shorter or longer than receptacle, and attached to body wall by posterior fibrils at level of first trunk constriction.**

**Table 3:** Morphometrics of adults of *Profilicollis altmani* from Peru, Chile, the USA.

Study site	Peru		Chile	USA
	Pasamayo, Lima	Ancỏn, Lima	Caleta Lenga	San Francisco
Host	*Larus belcheri*	*Larus belcheri*	*Larus pipixcan*	*Catoptrophorus semipalmatus* **
Source	Mateo et al. (1982)*	This study	Riquelme et al.(2006)*	Karl (1967)
Sample size	34 specimens	5MM, 11FF	9–16 specimens	10MM, 10FF
Males				
Total body length	15,600–16,400 (15,800)***	12,750–16,000 (14,500)	6000–21,700 (14,400)	**8500–16,600 (12,700)** ****
Trunk length	–	9375–12,000 (10,475)	4500–13,500 (11,100)	9800–14,170 (11,660)
Trunk width	1610–1850 (1700)	1250–2450 (1775)	1000–2000 (1300)	1510–3000 (2200)
Proboscis shape	Spheroid	Spheroid	Spheroid	Spheroid
Proboscis length	–	1025–1125 (1150)	500–1700 (1100)	**630–1220 (780)**
Proboscis width	1500–1870 (1660)	1200–1575 (1415)	–	**710–1230 (800)**
Rows of hooks	27–33	25–36 (30)	24–30 (28)	23–30 (27)
Hooks/row	13–15	11–13 (12)	13–15 (14)	10–15 (13)
Length of hooks				
Apical	44	38–42 (39)	–	30–50 (40)
Middle	33.5	37–43 (39)	–	30–60 (50)
Basal	54.7	50–58 (53)	–	40–60 (50)
Receptacle length	5740–7320 (6170)	**4125–5750 (5285)**	**1200–2600 (2400)**	**1910–3510 (2610)**
Receptacle width	460–530 (490)	**287–500 (417)**	–	310–740 (530)
Neck length	2830–3430 (3150)	**2375–3050 (2770)**	**1000–3000 (2100)**	**1360–2310 (1810)**
Neck width	430–480 (460)	350–575 (465)	200–500 (300)	310–490 (430)
Lemnisci length	2780–3600 (3100)	**2025–2925 (2667)**	1300–4000 (2400)	**1430–2400 (1610)**
Lemnisci width	290–360 (330)	**275–500 (417)**	–	–
Trunk spines				
Rows of spines	–	30–36 (32)	–	**25–30**
Spines/row	–	21–23 (22)	–	**10–16**
Spine length	–	25–30 (28)	–	–
Anterior testis length	960–1390 (1200)	**850–1200 (1055)**	300–1.700 **(0.800)**	**410–1110 (670)**
Anterior testis width	650–960 (790)	**600–800 (670)**	**100–800 (400)**	390–1010 (560)
Posterior testis length	1032–1390 (1250)	**875–1200 (1055)**	200–1600 **(800)**	410–1110 (670)
Posterior testis width	670–960 (800)	**488–700 (628)**	100–800 **(400)**	390–1010 (560)
Cement gland length	5520–6500 (5950)	**3375–5250 (4134)**	**200–600 (300)**	4000–5400
Cement gland width	90–150 (120)	65–150 (111)	–	–
Bursa length	**720** (average)	750–800 (775)	–	750–1100 (890)
Bursa width	**720** (average)	750–825 (787)	–	600–890 (630)
Females				
Total body length	16,500–21,600 (19,400)	10,000–23,000 (15,053)	10,700–28,500 (18,400)	**10,700–18,400 (15,400)**
Trunk length	–	6875–18,875 (11,267)	8000–18,500 (13,800)	**9800–14,170 (11,660)**
Trunk width	1560–1990 (1790)	1175–2200 (1602)	900–1700 (1800?)	1920–2630 (2310)
Proboscis shape	Spheroid	Spheroid	Spheroid	Spheroid
Proboscis length	–	675–1375 (956)	500–2000 (1200)	**630–1220 (780)**
Proboscis width	1540–1910 (1710)	**750–1575 (1127)**	600–2000 (1300)	**710–1230 (800)**
Rows of hooks	27–33	30–34 (32)	28–31 (29)	23–30 (27)
Hooks/row	13–15 **(usually 15)**	11–16 **(13)**	13–17 (14)	10–15 (13)
Length of hooks				
Apical	44	35–45 (38)	–	30–50 (40)
Middle	33.5	38–42 (39)	–	30–60 (50)
Basal	54.7	48–60 (55)	–	40–60 (50)
Receptacle length	6480–6720 (6600)	**4000–6500 (5539)**	**1100–4400 (3100)**	**1910–3510 (2610)**
Receptacle width	480–550 (520)	**200–550 (339)**	**–**	310–740 (530)
Neck length	2780–4200 (3490)	**1875–3825 (2953)**	**600–4000 (2500)**	**1360–2310 (1810)**
Neck width	380–550 (470)	425–625 (528)	300–600 (400)	310–490 (430)
Lemnisci length	3000–3890 (3460)	**1750–3755 (2603)**	2500–5200 (2600)	**1430–2400 (1610)**
Lemnisci width	312–530 (380)	**150–525 (336)**	–	–
Trunk spines				
Rows of spines	–	38–54 (47)	–	**25–30**
Spines/row	–	23–34 (28)	–	**10–16**
Spine length	–	22–30 (26)	–	–
Reproductive system length	5990	3675–8500 (5930)	–	–
Egg length	65.8–75.9 (71.3)	**52–60 (57)**	**50–70 (60)**	**60–70 (65)**
Egg width	24.0–37.6 (27.7)	**16–23 (19)**	**20–20 (20)**	**22–30 (26)**

* Described as *Polymorphus* (*Profilicollis*) *bullocki.*

** Karl (1967) reported 8 species of shore birds of California naturally infected with *P. altmani*, with *C. semipalmatus* being the most heavily infected species. His morphometrics were based on emended description of specimens obtained from his heavily infected experimental hosts, the duckling *Anas platyrhynchus* Linn. Common measurements of proboscis, hooks, neck, spine, receptacle and lemnisci were the same for both sexes.

*** Range (mean) in micrometers.

**** Bolded figures indicate atypically smaller measurements or counts.

### Males

Testes spheroid to ovoid, about equal, contiguous, pre-equatorial. Four unequal, tubular **cement glands usually terminating anteriorly at different levels occasionally reaching or overlapping posterior testis (****Figs. 29–30****). Four cement gland ducts emerging independently from cement glands posteriorly to join at juncture into bursa (****Figs. 31–33****). Prominent fibers and filaments appearing to connect cement gland ducts with posterior end of trunk from where others extending anteriorly (****Fig. 32****). Bursa bell-shaped with conspicuous but reduced rays and without apparent sensory structures (****Fig. 33****).**

### Female

**Reproductive system 40% length** of trunk with well-defined vagina, **2 sphincters, very long uterus, and small thin-walled uterine bell (UB) with few small nucleated UB cells (****Fig. 27****)**. **Four prominent horizontal nucleated cells at base of vagina and 4 more between sphincters (****Fig. 26****). Eggs fusiform with outer membrane appearing of hyaline components and inner membranes of fibrillar nature** and with slight polar prolongation of fertilization membrane (Fig. 28).

### Taxonomic summary

Host: Belcher’s gull, *Larus belcheri* (Vigors) (Aves: Laridae).

Other hosts: *Melanitta perspicillata* (Linn.) (type), *M. deglandi* (Bonaparte) (see Perry, [54]), *Larus argentatus* Pontoppidan (see Van Cleave, [75]), *Crocethia alba* (Pallas) (see Webster, [77]). Also see other hosts in Table 3 and the following 4 hosts listed by Riquelme et al. [56] from Chile: *Larus pipixcan* (Wagler), *L. dominicanus* Lichtenstein, *Numenius phaeopus* (Lin.), and *Podiceps occipitalis* Garnot. The other following 5 host species were also listed among 8 species recorded by Karl [42] from California: *Catoptrophorus semipalmatus* (Gmelin), *Cerorhinca monocerata* (Pallas), *Marila affinis* (Eyton), *Pelidna alpina* (Linn.), and *Phaeopus hudsonicus* (Latham).

Locality: Playa Pasamayo, Ancón (11°49′41″ S, 77°7′51″ W), Lima northern beach, Peru.

Other localities: See other localities in Table 3, among others along the Pacific and Atlantic coastlines of North and South America.

### Remarks

The adult populations of *P. altmani* compared in Table 3 show intraspecific variations that may be related to host species or geography. The 2 populations from *L. belcheri* from 2 different geographical locations along the Lima coastal line in Pasamayo and Ancon display the following variations. Our specimens from Ancon had smaller receptacle, neck, and lemnisci in both sexes, smaller testes and cement glands, and smaller eggs than specimens collected from the same host species from Pasamayo in the original description by Mateo et al. [47] (Table 3). Variations in size, especially when marked in certain structures, may be attributable geographical factors, assuming that host populations in these 2 collection sites do not mix.

Specimens from *L. pipixcan* in Chile (Table 1) had markedly smaller receptacle and neck in both sexes compared to those from both collections in Peru from *L. belcheri*, as well smaller mean testes measurements and considerably smaller cement gland length. The latter cement gland length measurement of 200–600 (300) from Chile [56] (page 468) is unreasonable and could not be verified by figures or other descriptions but was comparable to other measurements varying between 100 and 600 from 3 other sympatric host species (*L. dominicanus*, *Numenius phaeopus*, *Podiceps occipitalis*) examined by the same authors from Chile (their Table 1). Similarly, specimens from *Catoptrophorus semipalmatus* from California exhibit smaller size body, proboscis neck, neck, and lemnisci length in both sexes as well as number of trunk spines compared to specimens from Peru (Table 3). It is hard to compare Karl’s [42] specimens from California with others from elsewhere because he did not account for morphometric differences and counts of shared structures between sexes.

### Molecular analyses

Molecular analyses showed both phylogenetic trees gathered via ML and BI were mostly congruent (Fig. S1). The sequence of the cystacanth recovered from a specimen of *E. analoga* collected at Redondo Beach, California falls in the *P. altmani* clade (PP = 0.89; BS = 100; Fig. S1). Within this clade of *P. altmani*, no monophyletic group per geographic region or host is formed (see also [33, 46, 57, 59, 60]). Haplotypes of *P. altmani* show low genetic variation (average = 1.2%). *Profilicollis altmani* is sister to *P. botulus* (PP = 1; BS = 88); both species differ on average by 15%. The average genetic *p*-distance between the clades of *P. altmani* and *P. botulus* was 0.47. *P. chasmagnathi* and *P*. *novaezelandensis* are sister to each other (PP = 1; BS = 100). The average genetic *p*-distance between the clades of *P. altmani* and *P. chasmagnathi* was 0.35 (Fig. S1).

### Micropores

Micropores covered the whole trunk of male and female cystacanths (Fig. 13) as is commonly observed in adults in other species of acanthocephalans [16]. We did not create SEM images of adults; only whole-mounts were available. We assume, however, that adult *P. altmani* would also exhibit micropores on the cuticular surface of the trunk.

### Energy dispersive X-ray analysis (EDXA)

**Figures 41–43:**
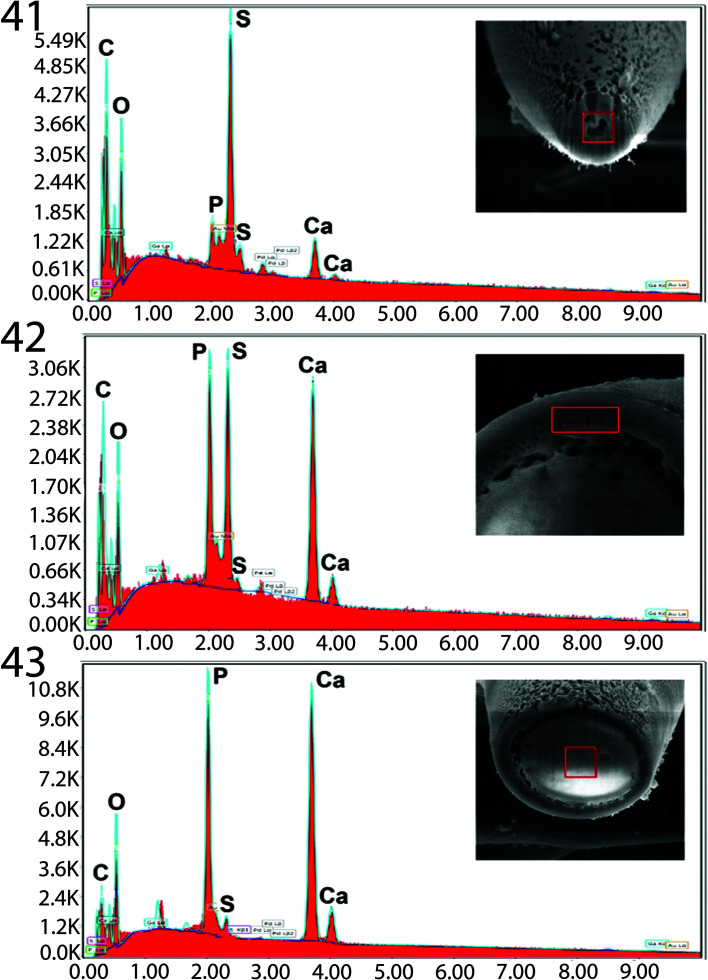
X-ray panels of elemental scans of *Profilicollis altmani* cystacanth hooks*.* See Table 4 for % weight of depicted elements. **41*.*** Scan of hook tip. Note the highest level of sulfur compared to the lower levels of phosphorous, magnesium, and calcium. Inset: hook tip. **42.** Scan of edge of mid-hook. Note the highest level of calcium and the moderate levels of sulfur and phosphorous. Insert: hook edge. **43.** Scan of center of mid-hook. Note the highest level of calcium and the lowest level of sulfur and magnesium. Insert: the center of a mid-hook. Center base of a longitudinal gallium cut showing typical levels of phosphorus, calcium and sulfur content. Insert: SEM of a longitudinal gallium cut hook.

The EDXA results of the hook cross-sections (Figs. 41–43, Table 4) of *P. altmani* cystacanths show a center core with high level of calcium and phosphorus surrounded by a sulfur-rich exterior. The EDXA spectra of the tip of the hook (Fig. 41) showed a significantly higher relative concentration of sulfur compared to the center core of the mid-hook cross-section (Fig. 43). The EDXA spectra of the edge of the mid-hook cross-section (Fig. 42) again shows the high-sulfur relative concentration observed in the tip of the hook (Fig. 41) as well as the high concentrations of calcium and phosphorus characteristic of the center core of the mid-hook cross-section (Fig. 43). The presence of sulfur, calcium, and phosphorus in the EDXA spectra obtained from the edge of the mid-hook cross-section is attributed to the proximity of the exterior shell to the center core. The relative WT% concentrations obtained by the TEAM software are reported in Table 4. It is worth noting that these reported WT% numbers should not be interpreted as compositional. They are, however, indicative of general compositional differences observed between the selected areas. EDXA for spines (Table 5) show low levels of all chemicals but a relatively higher level of calcium in anterior (6.34%) and middle (7.81%) with lowest levels in posterior-most spines. Similar analyses have not been conducted for other species of *Profilicollis*; thus, comparisons of the chemical profile of hooks and spines could not be made. A baseline for future comparisons is, however, established.

**Table 4:** Chemical composition of proboscis hooks of *Profilicollis altmani* cystacanths from *Emerita analoga* in Peru.

Element*	Hook tip	Edge of mid-hook	Center of mid-hook
Phosphorous (P)	2.66	9.65	17.26
Magnesium (Mg)	3.48	2.77	1.77
Sulfur (S)	14.82	11.11	1.60
Calcium (Ca)	5.13	22.18	39.31

* Palladium (Pd) and bold (Au) were used to count the specimens and gallium for the cross cut of the spines. These and other elements (C, O, N) common in organic matter are omitted. Data are reported in weight (WT%). Corresponding results are presented in Figures 41–43.

**Table 5:** Chemical composition of trunk spines of *Profilicollis altmani* cystacanths from *Emerita analoga* in Peru.

Element*	Single anterior spine	Several spines in anterior	Single middle spine	Posterior-most spine
Phosphorous (P)	3.93	2.61	5	1.15–1.34
Sulfur (S)	3.21	2.7	2.56	3.28–3.94
Calcium (Ca)	6.34	4.07	7.81	2.19–2.26

* Palladium (Pd) and gold (Au) were used to count the specimens and gallium for the cross cut of the spines. These and other elements (C, O, N) common in organic matter are omitted. Data are reported in weight (WT%).

### Histopathology

A series of selected sections (Figs. 37–40) represent the results of the histopathological study of the gull’s intestinal sections heavily infected with *P. altmani*. The invading worms were either attached to the gut wall or free in the lumen. There is prominent damage to the intestine where the worms interfaced with the mucosa and submucosa linings. Normal villi in the submucosa are greatly destroyed and disfigured out of shape (Figs. 37, 38). Sections of worms depict some internal anatomy such as lemnisci (Fig. 39). There is the potential for lumen blockage of the gull host gut when worms are present in excessive in numbers (Figs. 38, 39). Hemorrhaging and necrotic epithelial cells are visible around the invading worm with red blood cells free in the host lumen (Figs. 37, 40). The villi of the gull’s intestine are dramatically damaged and disfigured out of position with potential compromised absorption and subsequent hemorrhaging and epithelial connective tissue cell necrosis. Hemorrhaging of the blood vessels causes free blood cells and granulocytes to appear in interstitial spaces and in the lumen. Encapsulation has not been observed. The lesions, villi damage and worm number with potential lumen blockage will impact the feeding activity of the gull host.

## Discussion

### Geographically related morphological variations

Considering its wide range of host and geographical distribution, *P. altmani* exhibits a considerable range of morphometrical variability. The description of adults alone from many species of shore birds under different names in California [42, 54], Canada [75], Texas [77], Peru ([47] and this paper), and Chile [56] depict considerable variability. Table 3 further demonstrates extremes of morphological variations (see bolded figures) in some characters of adult populations including size of body, proboscis, receptacle, neck, lemnisci, testes, eggs and number of trunk spines in different hosts and geographical locations. The variability in cystacanths from various mole crab hosts in California, North Carolina, Peru, Chile, and Uruguay (Table 1) also demonstrates the relations between parasite characteristics and host and geography. These relationships have been previously reported for other species of acanthocephalans [16]. Examples of such relationships are discussed below.

A morphometric comparison between our Italian specimens of *Centrorhynchus globocaudaus* (Zeder, 1800) (Centrorhynchidae) and those from other geographical locations where comparative measurements and counts were reported was discussed by Amin et al. [19]. *Centrorhynchus globocaudaus* has been reported from many other locations in Asia, Africa, and Europe without taxonomic descriptions leaving their morphologic variability unaccounted for. Most measured specimens were collected from *Falco tinnunculus* Linn. (Falconidae) thus eliminating host species as a factor in observed differences in sizes. The specimens that we described had markedly longer proboscis hooks and larger receptacle, and a smaller male reproductive system (testes and cement glands) and lemnisci, especially in males compared to specimens from Ukraine, India, Egypt, Kyrgyzstan, Russia, Georgia, Armenia and former Asian Soviet Republics. Additionally, females from Ukraine and Kyrgyzstan had more proboscis hook rows and those from Ukraine had relatively larger eggs [19]. Such quantitative variations distinguishing the Italian population as a geographical variant had been previously demonstrated in the comparable case of *Mediorhynchus papillosus* Van Cleave, 1916 (Gigantorhynchidae) by Amin & Dailey [8]. Amin & Dailey [8] studied key taxonomic characteristics in various geographical populations of *M. papillosus,* which has a wide range of distribution in at least 73 species of birds outside of North and South America in Asia from Taiwan to the east into China, many of the former Soviet Republics, and to Eastern Europe to the west. Amin & Dailey [8] compared measurements of specimens from birds in Maryland, Colorado (their study material), Taiwan, Yakutia, Trans-Baikal, Lower Yansi River basin, the Volga basin and Oren Byreg (Orenburg), Ukraine, Bulgaria, China, and Brazil, and demonstrated distinct geographically-based variability, especially in the size of proboscis and its armature, neck, receptacle, and testes, that appeared related to geographical restrictions, intermediate and definitive host specificity and distribution, and host feeding behavior. The population variant of *C. globocaudaus* from Italy is, nevertheless, comparable to the east–west-intraspecific clinal variants of *M. papillosus* and could have been considered as distinct species but this notion is dismissed here also for the same reasons. The proposals of Amin and Dailey [8] support the observations of geographical morphometric variations in specimens of *Neoechinorhynchus* (*Hebesoma*) *personatus* Tkach, Sarabeev, Shvetsova, 2014 from the same host species, *Mugil cephalus*, in the Mediterranean and from the Black Sea ([20], Table 2), would point to intraspecific geographical variations. Van Cleave [73] made a vague reference to “geographical varieties” that was not supported by hard data. See [27, 28, 51] for more information.

Specimens of *Acanthocephaloides irregularis* Amin, Oguz, Heckmann, Tepe, Kvach, 2011 (Arhythmacanthidae) collected from *P. marmoratus* (Pallas) in Sukhyi Lyman off the Ukrainian coast were larger than those from the same host species collected from the gulf of Odessa [11]. Size differences of the trunk, proboscis, proboscis receptacle, lemnisci, and testes were attributed to the effect of factors in the geographical location affecting parasite growth and development [11]. Intraspecific variability related to geographical factors has also been demonstrated in *Rhadinorhynchus trachuri* Harada, 1935 (Rhadinorhynchidae). The observed differences in our *R. trachuri* specimens from Vietnam appear to represent intraspecific variations among Asian and American geographical populations, which may be affected by changes in feeding behavior [6].

Variability in proboscis hooks of *Acanthocephalus dirus* (Van Cleave, 1931) Van Cleave and Townsend, 1936 (Echinorhynchidae) populations in North America was studied by Amin [1] who noted that largest hooks were progressively longer in more northerly populations. The larger number of hooks per row (11–13) in the Mississippi population reported by Van Cleave [74] appears “to be characteristic to the extreme south” in the Upper Mississippi. The more northern populations were more variable and included specimens with as few as 6 hooks per row in males and 8 in females [1]. Bullock [23] also observed a larger number of proboscis hooks per row in *Acanthocephalus jacksoni* (Bullock, 1962) from New Hampshire than from Massachusetts. Similarly, Lincicome and Van Cleave [45] reported more and smaller hooks on the proboscis of *Leptorhynchoides thecatus*, possibly representing more than one species, from Canada than from the United States.

### Host related variations

All structures in our specimens of *Acanthogyrus* (*Acanthosentis*) *kashmirensis* Amin, Heckmann, Zargar, 2017 from *Schizothorax plagiostomus* Heckel and *S. labiatus* (McClelland) in the Sandran River in southern Kashmir are larger than those reported by Dhar [26] from his 4 other examined host species in the Jhelum River, and even counts of such features as the number of subcuticular nuclei are considerably greater ([14], page 458). The Sandran River is a tributary of the Jhelum River; both collections are considered sympatric and morphometric differences are attributed to host species. Host species may also have had an impact on the size of lemnisci, being equal or distinctly unequal in our specimens from A. *kashmirensis* and those of Dhar [26]. They were unequal only in specimens from *Bangana diplostoma* (Heckel), but equal in specimens from Dhar’s [26] 3 other species of fish.

The effect of host species on the size of acanthocephalan parasites has previously been reported in other species as well. See, for example, Amin and Redlin [7] who described considerably larger and more robust specimens of *Echinorhynchus salmonis* Müller, 1784 (Echinorhynchidae) from bloater, *Coregonus hoyi* Milner (Salmonidae), than from rainbow smelt, *Osmerus mordax* Mitchill (Osmeridae), collected from the same waters of Lake Michigan. Host species also affected corresponding differences in the size of proboscis, proboscis hooks, proboscis receptacle, lemnisci, testes, and cement glands as well as body form and differential growth rates in specimens from each host species. Linear regression analysis indicated that curves describing the growth pattern of these characters by worm length (age) were significantly different as a function of host species [7]. The larger worms recovered from *C. hoyi* invariably showed a higher regression coefficient compared to those from *O. mordax* in all characters. The taxonomic implications are vast and include the possible description of extremely variable populations from different host species (and geographies) as distinct species. We do not believe that this is an issue in our present investigation as long as we acknowledge that host species does account for the extreme differences in the morphometric characterization of the studied populations.

Comparative measurements of taxonomic characteristics of *Neoechinorhynchus* (*Neoechinorhynchus*) *johnii* Yamaguti, 1939 show that our specimens from 4 host species in Vietnam collectively had the largest size of trunk, proboscis, proboscis hooks, neck, and testes, and more cement gland nuclei compared to other specimens from India, Pakistan, and East China ([17], Table 2). When those measurements were broken down by host species ([17], Table 3), the relationship of host species to the size of taxonomically important structures became quite apparent.

Relevant studies on species of the genus *Acanthocephalus* and intraspecific variability in *Acanthocephalus dirus* [1, 2] explored the adaptability of acanthocephalans to a wide range of intermediate and definitive hosts affecting population dispersal and diversification. These examples, among others, point to plasticity in the Acanthocephala and the influence of biotic factors on their diversification. Our study, bearing out the possible existence of polymorphs of *P. altmani* is another demonstration of the variabilities within the Acanthocephala. This case of closely related, yet distinguishable, populations of one species can serve as a model for determining host and habitat specificity mechanisms.

### Molecular analysis

Our molecular results corroborate that despite morphological variability and host diversity, *P. altmani* displays low genetic variation and that there is no structure on the basis of hosts (intermediate and definitive) nor geography. Likely, the high mobility of the definitive hosts belonging to the family Charadriiformes, including the seagulls *L. dominicanus, L. modestus, C. maculipennis* and *L. pipixcan*, which are continuously migrating across oceans and hemispheres, contribute to the high dispersal of acanthocephalan eggs, causing genetic homogenization of populations and then, lack of phylogeographic structure [46, 57, 59, 60]. Future studies should evaluate if the observed pattern derived from the analysis of a mitochondrial gene, holds for more variable nuclear markers (e.g., SNPs). Finally, other studies should tackle the relationships among species of the family Polymorphidae, which are still unstable, suggesting that the limits of the genera that constitute this family need to be further evaluated.

### Micropores

The micropores of *P. altmani*, like those reported from other species of the Acanthocephala, are associated with internal crypts and vary in diameter and distribution in different trunk regions corresponding to differential absorption of nutrients. We have reported micropores in a large number of acanthocephalan species [38] and in a few more since, and demonstrated the tunneling from the cuticular surface into the internal crypts by TEM. Amin et al. [9] gave a summary of the structural–functional relationship of the micropores in various acanthocephalan species including *Rhadinorhynchus ornatus* Van Cleave, 1918, *Polymorphus minutus* (Goeze, 1782) Lühe, 1911, *Moniliformis moniliformis* (Bremser, 1811) Travassos (1915), *Macracanthorhynchus hirudinaceus* (Pallas, 1781) Travassos (1916, 1917), and *Sclerocollum rubrimaris* Schmidt and Paperna, 1978. Wright and Lumsden [79] and Byram & Fisher [24] reported that the peripheral canals of the micropores are continuous with canalicular crypts. These crypts appear to “constitute a huge increase in external surface area … implicated in nutrient uptake.” Whitfield [78] estimated a 44-fold increase at a surface density of 15 invaginations per 1 μm^2^ of *Moniliformis moniliformis* (Bremser, 1811) Travassos, 1915 tegumental surface. The micropores and the peripheral canal connections to the canaliculi of the inner layer of the tegument were demonstrated by transmission electron micrographs in *Corynosoma strumosum* (Rudolphi, 1802) Lühe, 1904 from the Caspian seal *Pusa caspica* (Gmelin) in the Caspian Sea (Figs. 19, 20 of [10]) and in *Neoechinorhynchus personatus* Tkach, Sarabeev, Shvetsova, 2014 from *Mugil cephalus* Linn. in Tunisia (Figs. 26, 29, 30 in [20]).

### Energy dispersive X-ray analysis (EDXA)

Our studies of acanthocephalan worms have usually involved X-ray scans (EDXA) of FIB-sectioned hooks and spines [35–37, 67]. Hooks and spines are evaluated for chemical ions with sulfur (S), calcium (Ca) and phosphorus (P) being the prominent elements. Sulfur is usually seen at the outer edge of large hooks and calcium and phosphorus are major ions in the base and middle of hooks where tension and strength are paramount for hook function. Large hooks play a major role for host tissue attachment. Results of the X-ray analysis of the FIB-sectioned hooks and spines (dual beam SEM) of *P. altmani* cystacanths show differential composition and distribution of metals in different hook and spine parts characteristic of that species. Hook tips of *P. altmani* showed the highest level of sulfur (14.82%) and lowest levels of calcium (5.13%) and phosphorus (2.66%) while the mid-hook center had highest level of calcium (39.31%) and phosphorus (17.26%) and lowest level of sulfur (1.6%). Similarly, in *Cavisoma magnum* (Southwell, 1927) Van Cleave, 1931 from *Mugil cephalus* in the Arabian Sea, unusually high levels of sulfur in hook tips (43.51 wt. %) and edges (27.46 wt. %) were found [15]. This element (sulfur) is part of the prominent outer layer of most acanthocephalan hooks and is a major contributor of the hardening process of this attachment structure. Our results are comparable to those of mammalian teeth enamel. The center and base of hooks of the same worms had negligible sulfur levels and contained mostly phosphorus and calcium, the two other essential elements for hook structure [15]. The chemical elements present in the hooks are typical for acanthocephalans [36, 37]. Note the moderate level of sulfur in the outer layer of the hook of *P. altmani,* which is different than in other acanthocephalans. The high sulfur content shows up in the outer edge of X-ray analysis of hooks (Table 4, 5, [15]). The hook center in mid cuts has a different chemical profile than the cortical layer (Table 4).

This is the first investigation of the chemical profile in spines of cystacanths of any species of acanthocephalan. The anterior, middle and posterior trunk spines of *P. altmani* show low levels of all chemicals but the calcium showed relatively higher levels than phosphorus and sulfur in all spines, reaching 7.81% in middle spines with the posterior spines having the lowest levels. These results suggest that trunk spines do contribute to the attachment and adhering to host tissue. There are no comparative studies of cystacanth spines to compare notes with and this study will serve as a reference line against which future studies can be evaluated.

X-ray scans analysis provide insight into the hardened components, e.g., calcium, sulfur, and phosphorus, of acanthocephalan hooks. The EDXA appears to be species-specific, as in fingerprints. For example, EDXA is shown to have significant diagnostic value in acanthocephalan systematics, e.g., *Moniliformis cryptosaudi* Amin, Heckmann, Sharifdini, Albayati, 2019 was erected based primarily on its EDXA pattern [18]. Our methodology for the detection of the chemical profile of hooks in the Acanthocephala has also been used in other parasitic groups including the Monogenea [64, 66] and Cestoda [65]. We also provide molecular data to explain and clarify our findings.

### Histopathology

Normal host intestinal tissue is evident in all sections showing prominent layers of the host intestine, especially the mucosa, sub-mucosa, and fibro-serosa. The sections are made from 4 heavily infected gulls, *L. belcheri* intestines packed with adult specimens of *P. altmani*. The mucosa is lined with simple columnar epithelial cells and goblet cells (Fig. 37). Host villi are compressed and distorted. There is a loss of the typical columnar epithelium lining (CEL) of the villi. The loss of the normal CEL of the mucosa and obstruction of the surface of the host mucosa will deprive it of nutrient absorption (Figs. 38–40). There is obstruction of the absorptive surface and blockage of the intestinal lumen due to the size of adult worms that often appear to occupy most of the host intestinal lumen. Worms have clearly invaded through the mucosal-submucosal layers causing observed cell necrosis, blood loss and destruction of the absorbing epithelial surface of the mucosa. Within the host, worms contained eggs and sperm (Fig. 40). The hemorrhaging is primarily due to capillary destruction of the host intestine. There is no evidence of encapsulation of worms by the host.

The histopathology results are similar to others described by Amin et al. [13] of *Moniliformis saudi* Amin, Heckmann, Mohammed, Evans, 2016 (Moniliformidae) in *Paraechinus aethiopicus* (Ehrenberg) [13] from Saudi Arabia and of *Centrorhynchus globirostris* Amin, Heckmann, Wilson, Keele, Khan, 2015 (Centrorhynchidae) in *Centropus sinensis* (Stephens) from Pakistan [39] as well as those of *Pomphorhynchus kashmirensis* Kaw, 1941 (Pomphorhynchidae) in *Schizothorax plagiostomus* Heckel [12] from Kashmir. Gonzales-Viera et al. [32] provided the only other histopathological study of *P. altmani* in another definitive host species, the gray gull, *Leucophaeus modestus* (Tschudi) from the Peruvian coast. Their sections were different from ours focusing on the attachment sites featuring proboscis invasion of the villi with adjacent central necrosis, inflammatory infiltrates, hyperplasia, granuloma in the submucosa, and peripheral dense fibrous and vascularized connective tissue. Gonzales-Viera ([32], page 115) further concluded that *P. altmani* “causes severe eosinophilic granulomatous enteritis in the gray gull.”

## Concluding remarks

This work brings into focus the importance of including studies of intraspecific variability in immatures and adults of acanthocephalans, elucidating developmental changes and adaptations. In addition to the utility of molecular profiles for comparative studies, the inclusion of Energy dispersive X-ray analyses of hooks and spines, as we present in this work, will contribute a valuable diagnostic tool often needed in taxonomic studies. When possible, histopathological studies will provide an additional dimension in studies of host-parasite relationships.

## Supplementary Materials

Supplementary material is available at https://www.parasite-journal.org/10.1051/parasite/2022005/olmTable S1. Species of acanthocephalans, host (intermediate (I) and definitive (D)), location, and GenBank accession number of the sequences used in the phylogenetic analysis.**Figure S1.**
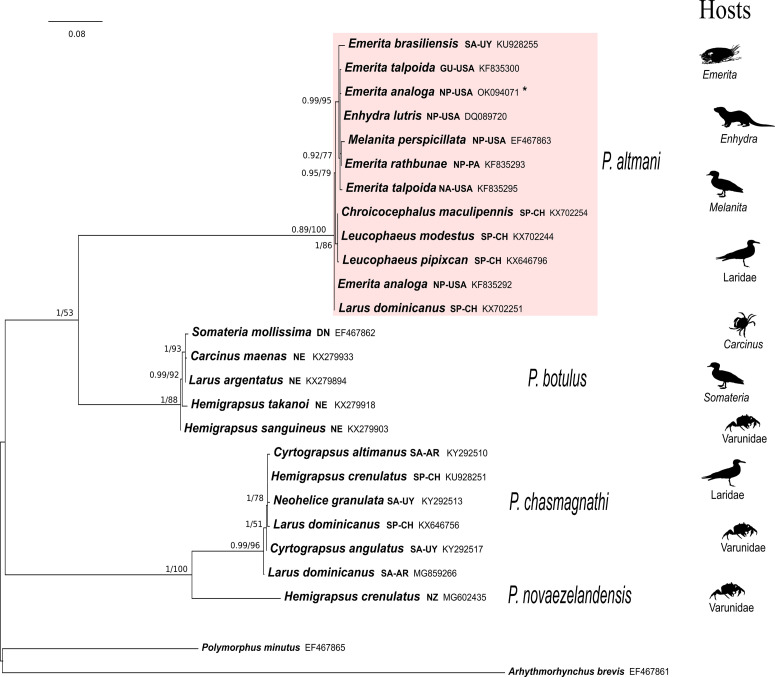
Genealogical relationships of haplotypes of the COI gene of specimens of the genus *Profilicollis* recovered in a Bayesian inference analysis. Numbers next to nodes refer to support values. Bayesian posterior probability values are shown left of the diagonal and Bootstrap proportions gathered in the Maximum Likelihood analysis (L_n_ = −2,762.492266) are shown right of the diagonal. GenBank accession numbers are included in the terminal labels. Animal silhouettes next to the tree indicate host of each species of *Profilicollis.***Figure S2.**
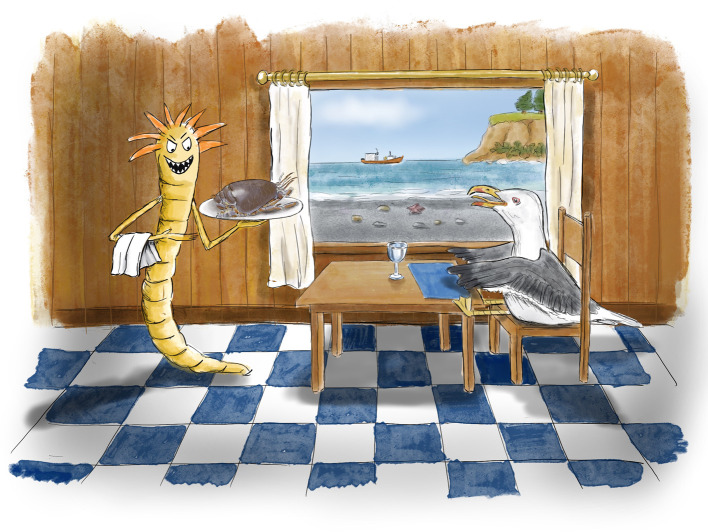
“Restaurante” – A painting by Denise de Solminihac, illustrating the research presented in this paper.

## Conflict of interest and ethical values

The authors declare compliance with relevant ethical values as well as no conflict of interest.
