# Supplementary material for: A comparative assessment of the morphology of Profilicollis altmani (Acanthocephala, Polymorphidae) from crustaceans and shore birds in Peru, with special notes on hook elemental analysis (EDXA), SEM imaging, histopathology, and molecular profile
Source: Parasite. 2022 Feb 22;29:9. doi: 10.1051/parasite/2022005 (PMC8862641; doi:10.1051/parasite/2022005)
Supplement: Supplementary file 1 — Species of acanthocephalans, host (intermediate (I) and definitive (D)), location, and GenBank accession number of the sequences used in the phylogenetic analysis. [file parasite-29-9-s1.pdf]

Supplementary material.

A comparative assessment of the morphology of *Profilicollis altmani* (Acanthocephala: Polymorphidae) from crustaceans and shore birds in Peru, with special notes on hook elemental analysis (EDXA), SEM imaging, histopathology, and molecular profile.

Omar M. Amin, Sara M. Rodríguez, Nataliya Rubtsova, Richard A. Heckmann, César Peña, Teresa Castro, Felipe Rivera, Guillermo D'Elía

Molecular methods

A sequence of a specimen *Profilicollis altmani* recovered from the mole crab *Emerita analoga* from Redondo Beach, California, USA, was gathered as follows. The tissue sample was digested overnight at 55ºC, and genomic DNA was isolated using a commercial extraction kit (Wizard® Genomic DNA Purification Kit, Promega, Madison, WI, USA). A fragment of the COI gene was amplified using the primers detailed by Folmer et al. [27] following the protocol of Amin et al. [16]. Amplicons were sequenced using an external sequencing service (Macrogen, Inc., Seoul, South Korea). The new DNA sequences was edited using Codon-Code (Codon Code Aligner, Dedham, MA, USA) and deposited in GenBank (OK094071; see Table S1).

The new COI sequence was aligned to a matrix with one representative of each haplotypic class of *Profilicollis altmani* and *P. chasmagnathi* found in both intermediate and definitive hosts from North and South America [33, 46, 57, 59, 60], *P. novaezelandensis* from the hairy-handed shore crabs *Hemigrapsus crenulatus* [34] and *P. botulus* from diverse intermediate and definitive hosts [29, 30]. These 23 sequences were downloaded from GenBank (Table S1). As such, a total of 24 sequences of *Profilicollis*

were analyzed. Sequences of *Polymorphus minutus* and *Arhythmorhynchus brevis*, which are closely related to *Profilicollis* [59], were used to form the outgroup.

Sequence alignment was done in Clustal as implemented in MEGA 7 [68] using the default parameter values. Observed genetic distances (*p*) between haplotype and sample pairs were calculated in MEGA 7. IQ-TREE [51] was used to select the model of nucleotide substitution (TPM3 + G4). Two methods of phylogenetic inference were implemented, maximum likelihood (ML) and Bayesian inference (BI). The ML analysis was conducted with IQ-TREE using the online implementation W-IQ-TREE (https:/iqtree.cibiv.univie.ac.at; [72]), with perturbation strength set to 0.5 and stopping rule set to 100. Clade support was calculated with 1,000 ultrafast bootstrap pseudo-replications (BS). The BI analysis was conducted with MrBayes 3.1 [62] with two independent runs with four heated and one cold Markov chains each. Runs lasted 20 million generations and parameters and trees were sampled every 1,000 generations. Model parameters were estimated in MrBayes. Convergence to stable log-likelihood values was checked by plotting log-likelihood values against generation time. The first 25% of the trees sampled were discarded as burn-in; remaining trees, all from the convergence zone, were used to compute a 50% majority rule consensus tree and to obtain posterior probability (PP) values for each clade.

Molecular results

The genealogical analysis showed that the sequence of the cystacanth recovered from a specimen of *E. analoga* collected at Redondo Beach, California, USA, falls in the *P. altmani* clade (PP = 0.89; BS = 100; Fig. S1). Haplotypes of *P. altmani* show low genetic variation; on average haplotype pairs differ by 1.2% (range 0–1.9%). Within this clade of

P. altmani no monophyletic group is formed by haplotypes recovered from each host, neither by developmental stage (cystacanths and adults; [33, 57, 59]). Similarly, the genetic variation of *P. altmani* is not geographically structured [33, 46, 57, 59, 60]. *Profilicollis altmani* is sister to *P. botulus* (PP = 1; BS = 88) in a relationship that has significant support only in the BI analysis (PP = 1; BS = 53; Fig. S1); both species differ on average by 15%. The clade of *P. botulus* is formed by cystacanths and adult worms extracted from brachyuran crabs, and adults from the Herring gull *Larus argentatus* Pontoppidan, 1763 and the Common eider *Somateria mollisima* (Linnaeus, 1758), collected in the Netherlands, Denmark and New Zealand. The average genetic *p*-distance between the clades of *P. altmani* and *P. botulus* was 0.47. *Profilicollis chasmagnathi* and *P. novaezelandensis* are sister to each other (PP = 1; BS = 100; Fig. S1). The average genetic *p*-distance between the clades of *P. altmani* and *P. chasmagnathi* was 0.35.

Table S1. Species of acanthocephalans, host (intermediate (I) and definitive (D)), location, and GenBank accession number of the sequences used in the phylogenetic analysis.

Species	Host	Location	GenBank access COI	References
Profilicollis altmani	*Emerita brasiliensis* (I)	South–Atlantic, Uruguay	KU928255	Rodríguez and D'Elía [57]
Profilicollis altmani	*Emerita talpoida* (I)	Gulf, USA	KF835300	Goulding and Cohen [33]
Profilicollis altmani	*Emerita analoga* (I)	North–Pacific, USA	**OK094071**	**This study**
Profilicollis altmani	*Enhydra lutris* (D)	North–Pacific, USA	DQ089720	García-Varela and Nadler [28]
Profilicollis altmani	*Melanitta perspicillata* (D)	North–Pacific, USA	EF467863	García-Varela and Ponce de León [29]
Profilicollis altmani	*Emerita rathbunae* (I)	North–Pacific, Panama	KF835293	Goulding and Cohen [33]
Profilicollis altmani	*Emerita talpoida* (I)	North–Atlantic, USA	KF835295	Goulding and Cohen [33]
Profilicollis altmani	*Chroicocephalus maculipennis* (D)	South–Pacific, Chile	KX702254	Rodríguez et al. [59]
Profilicollis altmani	*Leucophaeus modestus* (D)	South–Pacific, Chile	KX702244	Rodríguez et al. [59]
Profilicollis altmani	*Leucophaeus pipixcan* (D)	South–Pacific, Chile	KX646796	Rodríguez et al. [59]
Profilicollis altmani	*Emerita analoga* (I)	North–Pacific, USA	KF835292	Goulding and Cohen [33]
Profilicollis altmani	*Larus dominicanus* (D)	South–Pacific, Chile	KX702251	Rodríguez et al. [59]
Profilicollis botulus	*Somateria mollissima* (D)	Denmark	EF467862	García-Varela and Ponce de León [29]
Profilicollis botulus	*Carcinus maenas* (I)	Netherlands	KX279933	Goedknegt et al. [30]
Profilicollis botulus	*Larus argentatus* (D)	Netherlands	KX279894	Goedknegt et al. [30]
Profilicollis botulus	*Hemigrapsus takanoi* (I)	Netherlands	KX279918	Goedknegt et al. [30]
Profilicollis botulus	*Hemigrapsus sanguineus* (I)	Netherlands	KX279903	Goedknegt et al. [30]
Profilicollis chasmagnathi	*Cyrtograpsus altimanus* (I)	South–Atlantic, Argentina	KY292510	Rodríguez et al. [60]
Profilicollis chasmagnathi	*Hemigrapsus crenulatus* (I)	South–Pacific, Chile	KU928251	Rodríguez and D'Elía [57]
Profilicollis	*Neohelice*	South–Atlantic,	KY292513	Rodríguez et al.

chasmagnathi	*granulate* (I)	Uruguay		[60]
Profilicollis chasmagnathi	*Larus dominicanus* (D)	South–Pacific, Chile	KX646756	Rodríguez et al. [59]
Profilicollis chasmagnathi	*Cyrtograpsus angulatus* (I)	South–Atlantic, Uruguay	KY291517	Rodríguez et al. [60]
Profilicollis chasmagnathi	*Larus dominicanus* (D)	South–Atlantic, Argentina	MG859266	Lorenti et al. [46]
Profilicollis novaezelandensis	*Hemigrapsus crenulatus* (I)	New Zealand	MG602435	Hay et al. [34]
Polymorphus minutus	*Gammarus pulex* (I)	Dijon, France	EF467865	García-Varela and Ponce de León [29]
Arhythmorhynchus brevis	*Egretta thula* (D)	Guerrero, Mexico	EF467861	García-Varela and Ponce de León [29]
